# How and when do peer deviant behaviours link to adolescent academic performance?: Longitudinal moderated mediation analysis using a Chinese national sample

**DOI:** 10.1111/bjep.70097

**Published:** 2026-05-29

**Authors:** Yu Sun, Kaiji Zhou, Jacky C. K. Ng

**Affiliations:** ^1^ Department of Applied Social Sciences The Hong Kong Polytechnic University Hong Kong SAR China; ^2^ Mental Health Research Centre The Hong Kong Polytechnic University Hong Kong SAR China

**Keywords:** academic performance, Chinese students, deviant behaviours, deviant peers, educational expectations, peer effect

## Abstract

**Background:**

Previous research has associated peer deviant behaviours with lower academic performance in adolescents. However, the mediation and moderation mechanisms underlying this relationship, particularly the moderating role of educational expectations, require further exploration.

**Aims:**

This study aims to examine the longitudinal effect of peer deviant behaviours on academic performance among Chinese adolescents, as well as the mediating role of adolescents' own deviant behaviours and the moderating role of educational expectations.

**Sample:**

The study utilized a nationally representative two‐wave dataset from the China Education Panel Survey (CEPS, 2013–2015), including 8212 valid participants.

**Methods:**

A moderated mediation model was constructed to test the effect of peer deviant behaviours on academic performance and the underlying mechanisms.

**Results:**

Peer deviant behaviours negatively affected adolescents' academic performance over time. This relationship was mediated by adolescents' own deviant behaviours. Specifically, peer deviant behaviours increased adolescents' own deviant behaviours, which subsequently undermined academic performance. Educational expectations mitigated the impact of peer deviant behaviours on adolescents' own deviant behaviours, weakening the overall mediation effect. However, educational expectations could not mitigate the negative impact of adolescents' own deviant behaviours on their academic performance (the second stage of the mediation process).

**Conclusions:**

This study highlights the negative longitudinal effect of peer deviant behaviours on academic performance and pinpoints the stage at which educational expectations can mitigate this effect. These findings inform intervention strategies to address peer influence and foster academic success among adolescents.

## INTRODUCTION

Adolescent development has long been recognized as a critical issue in academic and policy circles. The intellectual and healthy development of youth during their formative years is linked to a nation's progress and long‐term prospects (Barro, 2001; Lutz et al., 2008). Within this context, academic performance serves as a primary indicator for assessing the standard and quality of education, especially in China, where a deeply ingrained cultural ethos has historically placed great emphasis on academic pursuits and educational attainment (Hannum & Park, 2007; Huang & Gove, 2015; Kipnis, 2011).

Adolescent academic development is influenced by various contextual factors, including school climate (Eccles & Roeser, 2011; Wang et al., 2017), family environment (Pomerantz & Wang, 2009; Tian & Jing, 2018; Zhao, 2013) and peer relationships (Hoxby, 2000; Rambaran et al., 2017). Among these, peers exert a particularly powerful influence, a phenomenon Coleman et al. (1966) conceptualized as the ‘peer effect’. This concept refers to the significant influence of interactions among individuals within the same social group on one's behaviour and serves as an essential factor in adolescent academic development. Since then, a substantial body of research has investigated peer effects in educational settings, underscoring its essential role (e.g., Hoxby, 2000; Rambaran et al., 2017; Sacerdote, 2011; Zimmerman, 2003).

Adolescence represents a critical transition period from childhood to adulthood, marked by increased susceptibility to external influences as adolescents construct their self‐identity and academic trajectories (Fredricks et al., 2004; Sacerdote, 2011). During this period, peers exert a salient influence on adolescents' behavioural adjustment and academic functioning (Fuligni et al., 2001; Rambaran et al., 2017; Rodkin & Ryan, 2012; Steinberg & Monahan, 2007). However, these studies are primarily grounded in Western individualistic contexts, leaving the necessity for cross‐cultural scrutiny. Furthermore, a similar dynamic has been observed longitudinally among Chinese children, where peer groups significantly influence both social functioning and academic achievement (Chen et al., 2008). Consequently, deviant peers can exert profound adverse impacts on adolescents. Therefore, understanding these peer dynamics within diverse cultural contexts is essential for educational stakeholders, as it informs interventions aimed at leveraging positive peer influences and mitigating detrimental effects within academic environments.

The present study focuses on the influence of peer deviant behaviours on adolescents' academic performance in China. To enhance our understanding of the potentially adverse effects of peer deviance, it is important to examine the underlying mechanisms and the protective factors that can buffer these effects. Therefore, the present research examines the moderated mediation process behind peer deviant behaviours, specifically investigating the potential mediator of adolescents' own deviant behaviours and the moderator of educational expectations.

### Influence of peer deviant behaviours on adolescents' academic performance

Peer deviant behaviours refer to adolescents' exposure to peers who engage in deviant behaviours, such as skipping class, stealing and fighting (Dishion & Tipsord, 2011; Monahan et al., 2009), which have been recognized as an important determinant of adolescents' academic performance. According to social contagion theory (Levy & Nail, 1993), behaviours, attitudes and emotions can spread through social networks via interpersonal interactions. Deviant peers' negative academic attitudes and poor academic behaviours can be transmitted within adolescent social networks, affecting adolescents' academic performance via poor study habits, impaired motivation, inadequate support and a poor learning environment (Jacobson & Burdsal, 2012; Sacerdote, 2011). Empirically, researchers have found that associating with peers who exhibit deviant behaviours, low academic achievement or high truancy levels may lead to a decline in adolescents' academic motivation and performance (Kang, 2007; Leka Peza, 2015; Rambaran et al., 2017). Conversely, affiliation with higher‐achieving peers tends to foster better academic outcomes (Kiss, 2013; Yang, 2021; Yang & Huang, 2020; Zhang et al., 2021).

However, many previous studies have failed to explicitly examine the mediation and moderation processes underlying the effects of peer deviance on academic outcomes (e.g., Gao & Xue, 2020; Han, 2022). Among the few studies that have examined these mechanisms, most have relied on cross‐sectional designs (e.g., Jiang, 2023), which provide limited evidence for establishing the mediating mechanisms and boundary conditions of the longitudinal deviant peer effects. More empirical investigations into the underlying mechanisms using a longitudinal design are necessary, especially addressing the long‐lasting nature of adolescents' behavioural and academic development (Chen & Vazsonyi, 2013; Dishion & Tipsord, 2011; Yu et al., 2025). The exposure to peers' deviant behaviours may have prolonged impacts on adolescents' deviant behaviours and academic performance over time (Dishion et al., 2010; Wang & Fredricks, 2014). Clarifying the mediating and moderating processes underlying such temporal effects can deepen our understanding of the cross‐time peer effects and inform more enduringly effective educational practices against adverse peer effects.

### The mediation role of adolescent deviant behaviours

As a contextual factor, peer deviant behaviours are likely to affect individuals' academic performance through modelling and reinforcement mechanisms. Drawing upon social learning theory (Bandura, 1977), peer deviant behaviours may lead to an increase in adolescents' deviant behaviours since adolescents may acquire certain behaviours through modelling and reinforcement during interactions with significant social groups. More specifically, Patterson's (1982) coercion theory suggests that antisocial behaviours are learned and maintained through coercive interaction cycles. This framework was extended by Dishion et al. (1996) into the concept of deviancy training, where adolescents mutually reinforce each other's rule‐breaking talk and behaviour through positive social reactions, such as laughter and approval.

In line with this work, empirical studies have shown that peer deviant behaviours are positively related to adolescents' own deviant behaviours (Helms et al., 2014; Prinstein & Wang, 2005; Shin et al., 2019). Consequently, the increase in deviant behaviours is likely to adversely impact their academic performance. Such behaviours (e.g., skipping class) typically disrupt adolescents' learning processes and divert them from normative academic behaviours, such as academic engagement and compliance with teachers' directives. Previous studies have also found that deviant behaviours negatively predict students' academic performance (Barriga et al., 2002; Henry et al., 2012; Wang & Fredricks, 2014). Taken together, peer deviant behaviours might lead to heightened deviant behaviours among adolescents, thus in turn resulting in lower academic performance. A recent study has shown that students' deviant behaviours mediated the relationship between peer deviant behaviours and academic performance (Jiang, 2023). However, due to the use of a cross‐sectional design, the temporal mediation process remains undetermined. As establishing a temporal effect is widely deemed important for examining peer influence among adolescents (Yu et al., 2025), a longitudinal design is needed to further investigate the cross‐time mediation role of adolescents' deviant behaviours and to better understand the temporal dynamics underlying these associations. We proposed Hypothesis 1:

*Adolescents' deviant behaviours mediate the longitudinal negative effect of peer deviant behaviours on adolescents' academic performance. Specifically, peer deviant behaviours positively predict adolescents' deviant behaviours, which are, in turn, negatively related to academic performance*.

Furthermore, these associations do not unfold in isolation. Recent evidence suggests that interactions with both parents and peers are important for adolescent development more broadly (Ding et al., 2026). Family social capital, reflected in relational family processes such as parent–child interaction, communication and engagement, has been recognized as an important resource for adolescents' behavioural adjustment and educational development (Byun et al., 2012; Coleman, 1988; Tan & Fang, 2023). Accordingly, such relevant background factors should also be considered when examining the link between peer deviant behaviours, adolescents' own deviant behaviours and their academic performance.

### The moderation role of educational expectations

Despite the aforementioned theoretical possibility and empirical findings, not all individuals are adversely influenced by peer deviant behaviours (Brechwald & Prinstein, 2011; Meldrum et al., 2013). Based on the risk‐buffering model, some individuals may possess protective factors or resilience that buffer them from the negative influences of deviant peers (Fergusson et al., 2007; Masten, 2001). Potential factors may alter the adverse influence of deviant peers on adolescents' own deviant behaviours, which, in turn, shape their academic performance. Although such protective resources may arise from multiple ecological contexts, the present study specifically examines adolescents' own educational expectations (Kao & Tienda, 1998; Ye et al., 2025) as the focal internal protective factor, while accounting for family relational factors (i.e., family social capital; Coleman, 1988) as relevant contextual covariates. In this way, we assess whether adolescents' own educational expectations retain a buffering role over family‐based resources.

Educational expectations, defined as an individual's anticipated level of future academic attainment (Kao & Tienda, 1998; Mickelson, 1990), play a crucial role in shaping adolescents' academic performance, motivation and future educational outcomes (Bates & Anderson, 2014; Domina et al., 2011; Ye et al., 2025). These expectations may buffer the effect of peer deviant affiliation by shaping adolescents' long‐term goals and self‐regulation. Drawing on self‐determination theory (Ryan & Deci, 2000) and the future time perspective theory (Lens et al., 2012), students with high educational expectations may have internalized motivation to prioritize academic achievement, leading to enhanced self‐discipline and resistance to behaviours that might jeopardize their future prospects, potentially mitigating the influence of peer deviant behaviours. Indirect evidence has shown that future orientation, a construct related to educational expectations (Zimbardo & Boyd, 1999), can weaken the association between peer deviant behaviours and problem behaviours (Beal & Crockett, 2010; Chen & Vazsonyi, 2013; Ouyang et al., 2023). However, direct evidence of whether educational expectations can mitigate the transmission of peer deviant behaviours into adolescents' own deviant behaviours and thus lower academic performance is sparse.

The risk‐buffering model is twofold: the protective‐stabilizing model (PSM) and the protective‐reactive model (PRM) (Sun et al., 2015). In PSM, a high level of a protective factor can completely neutralize the impact of a risk factor, such that the risk factor is significant only at low levels of the protective factor. In contrast, in PRM, the relationship between risk factors and outcomes is weaker yet still significant under high levels of a protective factor (Xie et al., 2018). However, due to the lack of previous empirical evidence, whether the moderating effect of educational expectations aligns with PSM or PRM remains unknown. Therefore, it is important to directly investigate the moderating effect. We proposed Hypotheses 2 and 3:

*Educational expectations mitigate the effect of peer deviant behaviours on adolescents' own deviant behaviours. Specifically, the positive relationship between peer deviant behaviours and adolescents' own deviant behaviours is weaker for adolescents with higher educational expectations*.

*Educational expectations mitigate the direct effect of peer deviant behaviours on academic performance. Specifically, the negative link between peer deviant behaviours and academic performance is weaker for adolescents with higher educational expectations*.

Meanwhile, we explored whether our data would support the PSM or PRM of the moderating effects.

In brief, a high level of educational expectations could be an internal protective asset against the external impacts of deviant peers on adolescents' behavioural and academic outcomes (i.e., buffering on the first‐stage mediation and direct path; see Figure 1). However, it remains unclear whether high educational expectations can still secure academic performance if adolescents themselves have already developed deviant behaviours (i.e., whether it mitigates the impact of one's own deviant behaviours on academic performance?) The latter is a more complex situation involving an internal tension between a ‘bright side of self’ (high educational expectations) versus a ‘dark side of self’ (one's own deviant behaviours). It remains empirically unclear which of these opposing internal forces—the protective effect of high expectations or the exacerbating effect of deviance—on academic performance will ultimately dominate.

**FIGURE 1:**
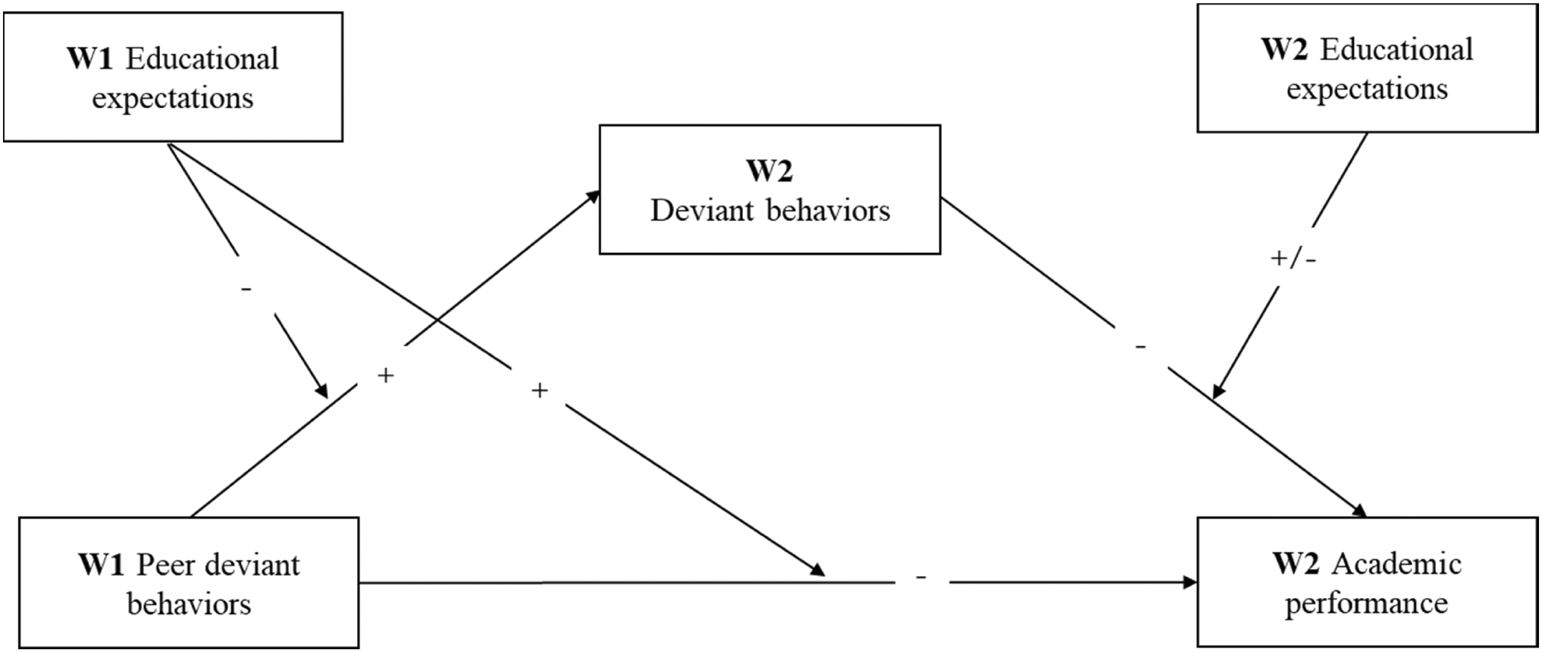
The conceptual framework of the moderated mediation model. Controlling for academic performance at W1 and other covariates.

On the one hand, as noted above, a high level of educational expectations helps maintain academic focus (Bates & Anderson, 2014; Domina et al., 2011). Therefore, adolescents with high educational expectations may manage to handle their studies (Wang & Fredricks, 2014), even if they, at the same time, have deviant behaviours. Nevertheless, the extent to which educational expectations would modify the relationship between adolescents' own deviant behaviours and academic performance remains empirically unclear. We proposed Hypothesis 4:

*Educational expectations mitigate the impact of adolescents' deviant behaviours on academic performance. Specifically, the negative link between adolescents' deviant behaviours and academic performance is weaker for adolescents with higher educational expectations*.

On the other hand, however, drawing on the cognitive dissonance perspective (Festinger, 1957; Stone & Cooper, 2001), students who have high educational expectations but also engage in deviant behaviours (themselves) may experience greater internal conflict. This may counteract the benefits of high educational expectations or even exacerbate academic decline, which means a possible negative moderation effect, as shown in Figure 1. Given the sparse existing empirical evidence and this alternative possibility, directly investigating the hypothesized buffering role of educational expectations in the impact of adolescents' own deviant behaviours (i.e., Hypothesis 4) is particularly necessary.

Taken together, it is possible that educational expectations can buffer the impact of peer deviant behaviours on adolescents' own deviant behaviours and the impact of adolescents' deviant behaviours on their academic performance. This implies a potential moderated mediation process where educational expectations mitigate the indirect effect of peer deviant behaviours on academic performance through one's own deviant behaviours. We proposed Hypothesis 5:

*Educational expectations mitigate the mediation effect of adolescents' own deviant behaviours on the association between peer deviant behaviours and academic performance. Specifically, the mediation effect of adolescents' deviant behaviours is weaker for adolescents with higher educational expectations*.

### The Chinese context

This study examines the link between peer deviant behaviours and adolescent academic performance within the distinctive context of China. While peer influence is a well‐established correlate of academic outcomes in Western literature, its manifestation in China is likely shaped by the nation's unique sociocultural dynamics. First, the Chinese educational context is characterized by a high‐stakes academic system underpinned by sociocultural values that place a profound emphasis on educational achievement (Hannum & Park, 2007; Kipnis, 2011). Within this context, academic success, particularly in the national college entrance exam, is regarded as an important gateway to social mobility. This fuels intense competition and stress among students, a pressure systematically reinforced by teachers, parents and a broader social context. It is within this pressurized educational environment that the established mechanism of ‘deviancy training’ (Dishion et al., 1996) may present a distinct character: deviant peer groups in China may particularly reinforce school‐specific resistance, such as academic disengagement, as a direct response to systemic pressures, thereby making the peer deviant influence widely relevant and critical for Chinese students' academic outcomes. Emerging empirical evidence from China corroborates this link, indicating that heightened academic pressure is intertwined with adolescents' deviance and subsequent declines in academic performance (Jiang et al., 2022; Jiang & Zhou, 2025). Moreover, under the intense competition in the Chinese educational system, high educational expectations may also entail a risk of heightened psychological pressure (Zhou et al., 2021). It is therefore uncertain whether educational expectations can retain their protective effect against peer deviancy among Chinese adolescents, an issue that warrants direct investigation.

Second, Chinese adolescents are exposed to various evolving social values, influenced by socioeconomic changes, such as urbanization and economic reforms, which have weakened traditional societal norms (Greenfield, 2009; Li et al., 2010; Yan, 2010). The absence of an established and unified social value system may induce normative confusion among youth (Jensen et al., 2011; Schwartz et al., 2005), predisposing them to the influences of deviant peers when they are forming their personal values and worldviews (Brechwald & Prinstein, 2011). Additionally, the large‐scale rural–urban migration in contemporary China has challenged traditional family and community ties (Fan et al., 2010; Lu, 2012; Wu & Zhang, 2015; Xu & Xie, 2015). It may leave adolescents feeling isolated from stable support systems and increase their need for belonging (Baumeister & Leary, 1995; Gao et al., 2010), which potentially drives them to join deviant peer groups for social connection, thus increasing exposure to peer deviant behaviours (Dishion et al., 1994; Véronneau & Dishion, 2011).

Therefore, by uncovering the mechanisms through which peer deviance affects academic outcomes and the potential protective function of educational expectations, this research aims to contribute to a more nuanced understanding of peer effects in the Chinese context.

## THE PRESENT STUDY

Although the association between peer deviant behaviours and adolescents' academic performance has been extensively documented, the mediation and moderation mechanisms are less clear, especially among Chinese adolescents. To address this gap, this study proposes and examines a moderated mediation model linking peer deviant behaviours to the academic performance of Chinese adolescents, with a specific focus on adolescents' own deviant behaviour as a mediator and their educational expectations as a potential protective factor that buffers these associations (see Figure 1 for the conceptual model). In addition, as noted above, this analysis incorporated family social capital indicators as covariates to account for essential family relational resources. In alignment with the existing empirical framework (Tan & Fang, 2023; Yang & Dai, 2019), these indicators encompassed frequency in parent–child interactions, parent–child discussion about school experiences, quality of parent–child relationship, parental engagement with the child's educational activities and parental expectations for the child's academic attainment. By testing this model among Chinese adolescents, we aimed to situate core constructs from Western peer effect theories (e.g., Dishion et al., 1994; Patterson, 1982) within the Chinese context, thereby exploring their applicability and potential boundary conditions. This research utilizes longitudinal, nationally representative data to rigorously examine the temporal relationships among these variables.

## METHODS

### Participants and design

This study utilized secondary data from the China Education Panel Survey (CEPS) at http://ceps.ruc.edu.cn/, a nationally representative, large‐scale longitudinal survey conducted by the National Survey Research Center (NSRC) at Renmin University of China. The CEPS employed a rigorous multi‐stage sampling strategy with probability proportional to size (PPS) methods. At the primary stage, 28 county‐level administrative units were probabilistically selected. Within each sampled county, four junior high schools offering Grade 7 and/or Grade 9 were systematically involved. The third stage randomly selected two classes per eligible grade in each school, with all students in attendance on the survey date administered the questionnaire. The baseline wave was carried out during the 2013–2014 academic year and included 19,487 students of Grade 7 and Grade 9 from 438 classes in 112 schools. The first follow‐up investigation during the 2014–2015 academic year tracked 10,279 students in Grade 8 (they were in Grade 7 at the time of the baseline survey). The number of students who were successfully tracked was 9449 (retention rate = 91.9%).

The dataset consists of the first wave of data collected during the 2013–2014 academic year (Wave 1) and the follow‐up wave of the 2014–2015 academic year (Wave 2). Using school ID and class number, the sub‐datasets for student and school surveys were merged to integrate student information with school and class information. Consistent with prior practice based on CEPS (e.g., Yang & Dai, 2019; Zhang, 2024), the analytical sample includes students who participated in both survey waves and provided complete data on the focal variables (*N* = 8212). The demographic characteristics of the working sample are shown in Table 1.

**TABLE 1:** Sample characteristics and descriptive statistics.

*N* = 8212		*n* (%)	Min	Max
Covariates (Wave 1)				
Gender	Male	4076 (49.6)		
	Female	4136 (50.4)		
Residency status	Local Hukou	6681 (81.4)		
	Non‐local Hukou	1531 (18.6)		
Only child	The only child	3714 (45.2)		
	Not the only child	4498 (54.8)		
Boarding	Living at school (weekdays)	2500 (30.4)		
	Not living at school	5712 (69.6)		
		Mean ± SD		
Family economic status		2.820 ± .591	1	5
Parental educational level		4.631 ± 2.027	1	9
Parental engagement		2.422 ± 1.039	1	4
Parental expectations		2.982 ± .835	1	4
Parent–child relationship		2.694 ± .448		
Parent–child interaction		3.776 ± 1.150	0	6
Parent–child discussion		2.001 ± .566	0	3
Focal variables (Waves 1 and 2)				
Academic performance (converted score)	Wave 1	70.709 ± 8.256	16.892	97.977
	Wave 2	70.603 ± 8.492	16.993	95.231
Peer deviant behaviours	Wave 1	1.088 ± .233	.143	3
Deviant behaviours	Wave 2	1.531 ± .456	.3	5
Adolescent educational expectations	Wave 1	7.027 ± 1.627	1	9
	Wave 2	6.805 ± 1.600	1	9

### Measures

#### Peer deviant behaviours (wave 1)

In CEPS, the extent to which a student's close friends exhibit deviant behaviours was measured using a 7‐item scale describing deviant behaviours of close friends, such as skipping classes (see Appendix S1 for the measure items). Each item was rated on a three‐point scale, ranging from 1 = *none of them* to 3 = *most of them*. The final score was calculated as the mean of the seven items, where a higher mean score indicates more deviant peer behaviours in the student's close friends. In this research, the scale demonstrated good reliability (Cronbach's alpha = .874, McDonald's omega = .877).

#### Deviant behaviour (wave 2)

A 10‐item scale measured the frequency of deviant behaviours exhibited by the student, such as skipping classes (see Appendix S1 for items).^1^ Responses were measured on a 5‐point scale from 1 = *never* to 5 = *always*. The adolescent's deviant behaviour score was calculated as the mean of the ten items. A higher mean score reflects a greater frequency of self‐reported deviant acts. The scale showed acceptable reliability (Cronbach's alpha = .816, McDonald's omega = .820).

#### Educational expectations (waves 1 and 2)

Educational expectations, reflecting the highest level of education the student expects to receive, were measured by a single‐item, 9‐point scale assessing aspirations for future educational attainment, ranging from 1 = *dropout for now* to 9 = *doctoral degree*. The same item was used in both Wave 1 and Wave 2 surveys. Responses to ‘does not matter’ were excluded.

#### Academic performance (waves 1 and 2)

The academic performance at Wave 1 and Wave 2 was indicated by the midterm examination results of the three ‘major subjects’, namely, Chinese, mathematics and English, of junior high school students in the 2013–2015 academic year (Wave 1) and the 2014–2015 academic year (Wave 2), respectively. The examination scores were provided by the surveyed schools, which were standardized based on the school unit, with a mean of 70 points and a standard deviation (SD) of 10 points. A composite variable of academic performance was created by averaging the standardized scores of the three subjects. In this research, academic performance at Wave 2 is the outcome variable, with academic performance at Wave 1 serving as the baseline control variable.

#### Family social capital (wave 1)

Following Coleman's (1988) conceptualization, prior empirical operationalizations (e.g., Byun et al., 2012; Dufur et al., 2013; Israel et al., 2001; Shahidul et al., 2015), and especially CEPS‐based operationalizations (e.g., Tan & Fang, 2023; Yang & Dai, 2019), we included five family social capital indicators from Wave 1: (1) parent–child interaction was assessed by the frequency of joint activities with parents (6 items; 6‐point scale); (2) parent–child discussion was measured by the frequency of discussing school‐related issues with the mother and father (5 items for each parent; 3‐point scale); (3) parent–child relationship was indexed by adolescents' perceived closeness with the mother and father (2 items; 3‐point scale); (4) parental engagement was measured by parental supervision and guidance regarding the adolescent's studies and homework (2 items; 4‐point scale); (5) parental expectations for academic performance were assessed with a single item on parents' expectations regarding the adolescent's school achievement (4‐point scale). See Appendix S1 for the full list of items. Reliability for the multi‐item measures was acceptable, with Cronbach's alpha ranging from .641 to .826 and McDonald's omega ranging from .747 to .825. A mean score was calculated for each multi‐item indicator, with higher scores indicating greater family relational resources. These indicators were included in the analyses as background covariates.

#### Other covariates

In addition to the family social capital indicators and academic performance at Wave 1, this study controlled for gender (1 = *female*, 0 = *male*), family economic status (from 1 = *very poor* to 5 = *very rich*), highest parental education level (comparing the education levels of the student's father and mother and taking the relatively higher value: from 1 = *no education* to 9 = *postgraduate and above*), only‐child status (1 = *yes*, 0 = *no*), boarding (1 = *yes*, 0 = *no*), school ranking (ranking in the county/district: 1 = *medium and below*, 2 = *above medium* or 3 = *best*) and class ranking (ranking within the grade: 1 = *below medium and worst*, 2 = *medium and above medium* or 3 = *best*). These covariates have been widely used in previous research in this field (Huang et al., 2023; Jiang, 2023; Yang, 2021; Yang & Huang, 2020). Additionally, as noted above, adolescents' and their peers' geographic mobility may complicate peer effects. Therefore, we also included migration‐related variables in CEPS: residency status (*local hukou* = 1, *non‐local hukou* = 0), school type (hukou) (four degrees of local student proportion: <50% [1], 50%–70% [2], 70%–90% [3], >90% [4]) and class type (four degrees of local student proportion: <50% [1], 50%–70% [2], 70%–90% [3], >90% [4]).

### Statistical analyses

We first examined a longitudinal mediation model with peer deviant behaviours at Wave 1 (W1) as the independent variable, deviant behaviours at Wave 2 (W2) as the mediator, and academic performance at Wave 2 (W2) as the dependent variable, with adolescents' academic performance at W1 controlled to account for its autoregressive effect over time. The autoregression for the W2 mediator (prediction from W1 deviant behaviours to W2 deviant behaviours) was not included since there is no corresponding measure of deviant behaviours in W1 comparable with the deviant behaviours measure used for W2 in the CEPS dataset. Next, we constructed the moderated mediation model by specifying the educational expectations at W1 as the moderator in the first‐stage mediation pathway and specifying the educational expectations at W2 as the moderator in the second‐stage mediation pathway (as shown in Figure 1). The mediation analysis, moderation analysis and moderated mediation analysis were conducted with the PROCESS macro in SPSS version 29.0 (bootstrap resampling = 5000). The Johnson–Neyman plot illustrating the moderating effect was created using R version 4.4.1.

## RESULTS

### Descriptive statistics and correlation analyses

The results of descriptive statistics are shown in Table 1. The results of the bivariate correlation analysis of all variables are shown in Table 2.

**TABLE 2:** Pearson correlations among the study variables.

	1	2	3	4	5	6	7	8	9	10	11	12	13	14	15	16	17	18	19	20	21
1. W1_EE	1																				
2. W2_EE	.534***	1																			
3. W2_DB	−.156***	−.229***	1																		
4. W1_PDB	−.126***	−.131***	.210***	1																	
5. W1_AP	.334***	.374***	−.120***	−.112***	1																
6. W2_AP	.338***	.404***	−.159***	−.125***	.777***	1															
7. W1_PCI	.178***	.178***	−.151***	−.024*	.033**	.043***	1														
8. W1_PCD	.195***	.176***	−.120***	−.082***	.118***	.122***	.358***	1													
9. W1_PCR	.107***	.097***	−.108***	−.082***	.064***	.075***	.259***	.315***	1												
10. W1_PEG	.088***	.059***	−.088***	−.034**	−.075***	−.078***	.352***	.254***	.157***	1											
11. W1_PE	.322***	.306***	−.084***	−.069***	.415***	.395***	.078***	.129***	.081***	.035**	1										
12. W1_gender	−.100***	−.107***	.150***	.144***	−.237***	−.247***	−.007	−.078***	−.014	.031**	−.069***	1									
13. W1_SES	.109***	.107***	−.046***	−.016	.009	.006	.215***	.086***	.057***	.087***	.018	−.001	1								
14. W1_OC	−.164***	−.156***	.102***	.041***	−.018	−.009	−.236***	−.135***	−.063***	−.179***	−.008	−.055***	−.167***	1							
15.W1_migration	−.038***	−.023*	.023*	.018	−.017	−.004	.030**	−.042***	−.058***	.003	−.006	.015	.045***	.086***	1						
16. W1_boarding	.090***	.099***	−.127***	−.055***	−.029**	−.020	.204***	.018	−.054***	.181***	−.036***	−.007	.186***	−.296***	.154***	1					
17. W1_PHE	.256***	.241***	−.111***	−.052***	.063***	.061***	.270***	.161***	.060***	.177***	.054***	−.009	.223***	−.370***	−.046***	.248***	1				
18. W1_ST	.013	.009	.027*	−.026*	.015	−.006	−.125***	.032**	.079***	−.055***	.029**	−.012	−.127***	.124***	−.465***	−.318***	−.084***	1			
19. W1_SR	.197***	.185***	−.086***	−.078***	.000	−.005	.143***	.131***	.076***	.064***	.036***	−.023*	0.131***	−.173***	−.143***	.076***	.239***	.244***	1		
20. W1_CT	.005	−.005	.042***	−.023*	.024*	.006	−.142***	.024*	.079***	−.062***	.028*	−.010	−.146***	.147***	−.482***	−.354***	−.110***	.905***	.194***	1	
21. W1_CR	.074***	.074***	−.064***	−.065***	.088***	.074***	.046***	.062***	.031**	.012	.021	−.035**	.064***	−.086***	−.038***	.029**	.069***	.058***	.018	.043***	1

*Note*: *** *p* < .001, ** *p* < .01, * *p* < .05.

Abbreviations: AP, academic performance; CR, class rank; CT, class type (migrants' proportion); DB, deviant behaviours; EE, adolescent educational expectations; OC, only child; PCD, parent–child discussion; PCI, parent–child interactions; PCR, parent–child relationship; PDB, peer deviant behaviours; PEE, parental educational expectations; PEG, parental engagement; PHE, parental highest educational level; SES, subjective socioeconomic status; ST, school type (migrants' proportion), SR, school rank; W1, Wave 1; W2, Wave 2.

### Mediation analysis

A mediation model (Model 4 in the PROCESS macro; bootstrapping resampling = 5000) was constructed to examine the mediation effect of W2 deviant behaviours in the link between W1 peer deviant behaviours and W2 academic performance. The results showed that the total effect (*B* = −.958, *SE* = .224, *p* < .001, 95% CI = [−1.397, −.519], *β* = −.026), direct effect (*B* = −.637, *SE* = .228, *p* < .01, 95% CI = [−1.084, −.191], *β* = −.017) and indirect effect (Estimate = −.321, *SE* = .060, 95% CI = [−.445, −.211], *β* = −.009) were significant after controlling for W1 academic performance and other covariates as outlined above. Specifically, peer deviant behaviours at W1 positively and significantly predicted adolescents' own deviant behaviours at W2 (*B* = .379, *SE* = .021, *p* < .001, 95% CI = [.338, .420], *β* = .200), which was in turn negatively related to adolescents' academic performance at W2 (*B* = −.847, *SE* = .117, *p* < .001, 95% CI = [−1.078, −.617], *β* = −.048).

The mediation effect accounts for 33.5% of the total effect, with the corrected *R* ^2^ of .710 in the process model (Ng, 2024). This finding indicates that peer deviant behaviour could significantly predict academic performance among adolescents through the mediation of adolescents' deviant behaviours, supporting Hypothesis 1.

### Moderated mediation analysis

A moderated mediation model was constructed with Model 22 in the PROCESS macro to estimate the original hypothesized paths (as shown in Figure 1). Variables for generating interaction terms were all grand‐mean centred. There were non‐significant moderating effects of W2 educational expectations on the second stage of the mediation (i.e., the path from W2 deviant behaviours to W2 academic performance) and the non‐significant moderating effect of both waves of educational expectations on the direct effect (*p* > .1). After removing the non‐significant moderator (W 2 educational expectations) and non‐significant moderating effects, we obtained the final moderated mediation model as shown in Figure 2.

**FIGURE 2:**
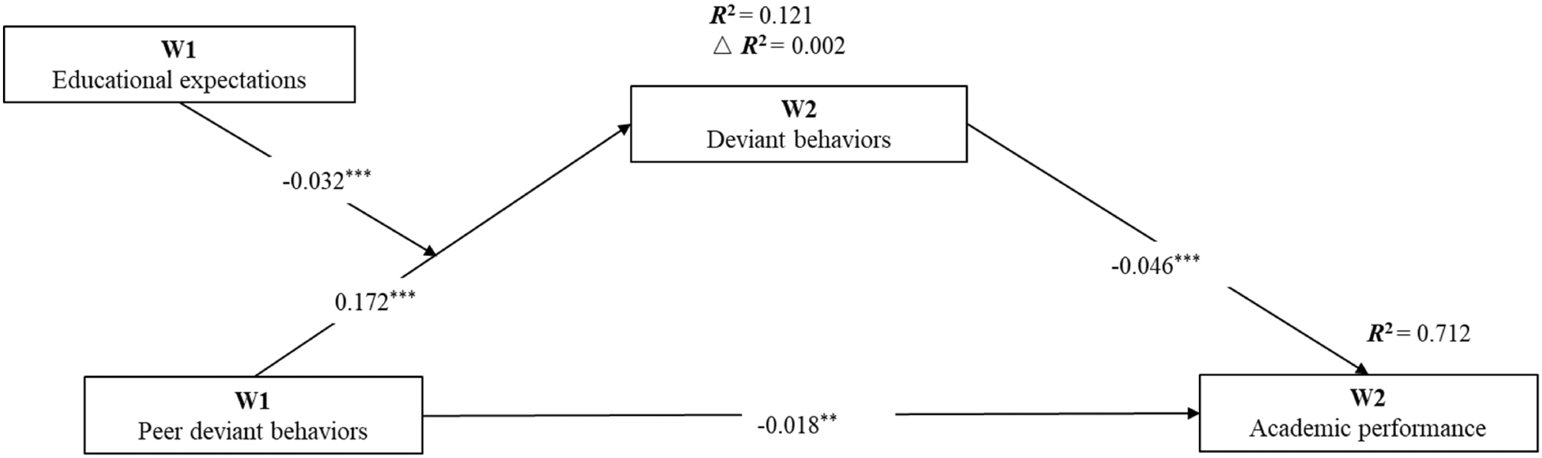
Final moderated mediation model. Standardized coefficients (*β*s) are presented. Controlling for W1 academic performance, gender, family economic status, parental highest educational level, only child, residency status, boarding, school type, school rank, class type, class rank and family social capital indicators. W1, Wave 1; W2, Wave 2; ***p < .001, **p < .01.

The final moderated mediation model was constructed with Model 7 of the PROCESS macro. As shown in Figure 2, W1 peer deviant behaviours could significantly and negatively predict W2 academic performance through the mediation role of W2 deviant behaviours.

The results of the moderated mediation analysis are shown in Table 3. Educational expectations negatively moderated the positive link between Wave 1 peer deviant behaviours and Wave 2 deviant behaviours, indicating that educational expectations could mitigate the adverse predictive effect of peer deviant behaviours on adolescents' own deviant behaviours, supporting our Hypothesis 2. However, Hypotheses 3 and 4 were not supported.

**TABLE 3:** Results of moderated mediation analysis.

Regression equation		Fitting index			Coefficient				95% CI	
Outcome	Predictor	*R*	*R* ^2^	*F*	*B*	*SE*	*t*	*β*	LL	UL
W2 DB		.319	.121	56.614***						
	W1 PDB				.339	.022	15.395***	.172	.296	.392
	W1 EE				−.012	.003	−3.613***	−.045	−.019	−.006
	PDB × EE				−.037	.010	−3.940***	−.032	−.057	−.019
W2 AP		.844	.712	1064.280***						
	W1 PDB				−.641	.228	−3.336**	−.018	−1.088	−.194
	W2 DB				−.851	.118	−7.227***	−.046	−1.081	−.620

	W1 EE conditions	*B*	*SE*	*t*	*β*	LL	UL
Simple slopes	−1 SD	.401	.023	17.479***	.205	.356	.446
	Mean	.339	.022	15.395***	.172	.296	.382
	+1 SD	.277	.031	9.020***	.140	.217	.337

*Note*: The results for covariates (as outlined above) and intercepts are not displayed in the table. ***p < .001, **p < .01.

Abbreviations: AP, academic performance; DB, deviant behaviors; EE, educational expectations; LL, 95% bootstrap confidence interval lower limit of *B;* PDB, peer deviant behaviors; UL, 95% bootstrap confidence interval upper limit of *B*.

The results of simple slope analysis are shown in Table 3 and Figure 3. The predictive effect of peer deviant behaviours on deviant behaviours at high educational expectations (mean + 1 SD) was significantly weaker than that at low educational expectations (mean − 1 SD) (*β* contrast _high vs. low_ = −.065, *p* < .05).

**FIGURE 3:**
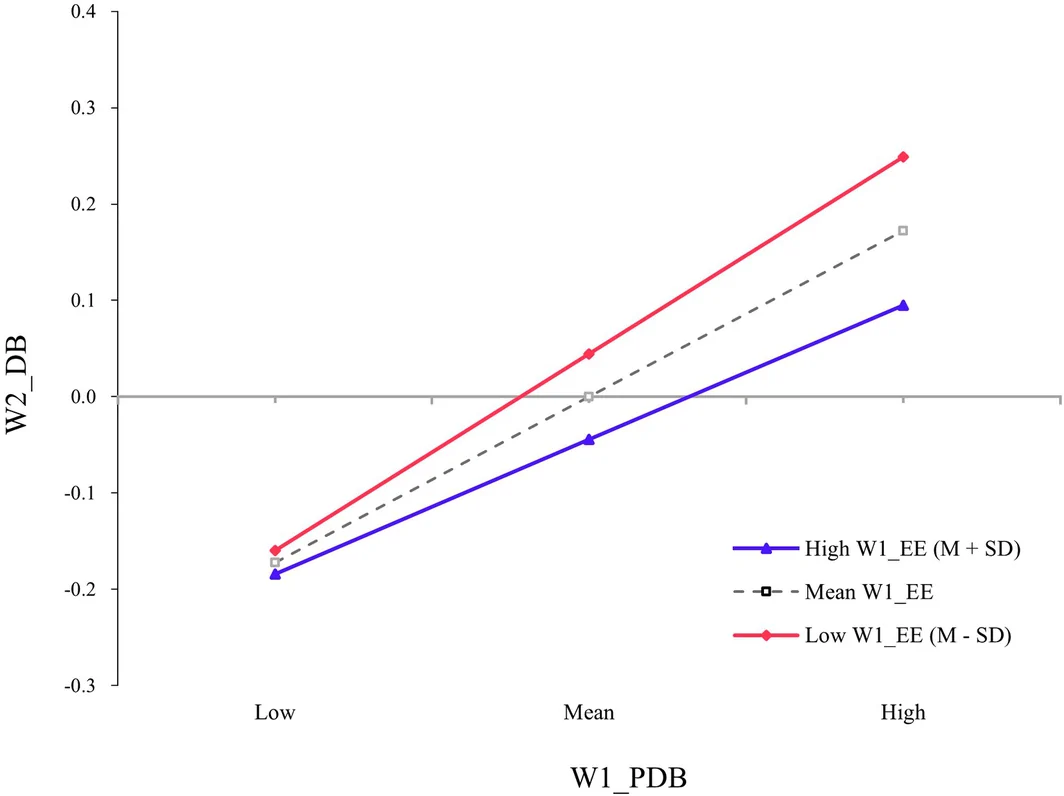
The simple slope plot of the interaction between peer deviant behaviours and educational expectations in predicting adolescents' deviant behaviours. DB, deviant behaviours; EE, educational expectations; W1, Wave 1; W2, Wave 2.

Figure 4 shows the Johnson–Neyman plot, which helps identify the ranges of moderator values within which the effect of the independent variable was significant or non‐significant (Lin, 2020). This can pinpoint the potential threshold at which the moderator (educational expectations) can neutralize the adverse impact of peer deviant behaviours. However, as shown in Figure 4, the significance zone of the effect of peer deviant behaviours covered the whole range of educational expectations, while the non‐significance zone occurred only when educational expectations reached a very high level (>11.302), exceeding the variable range (1–9). This result indicates that although educational expectations significantly reduced the effect of peer deviant behaviours (as noted above; *β* = −.032, *p* < .001), they could not completely neutralize the effect of peer deviant behaviours.

**FIGURE 4:**
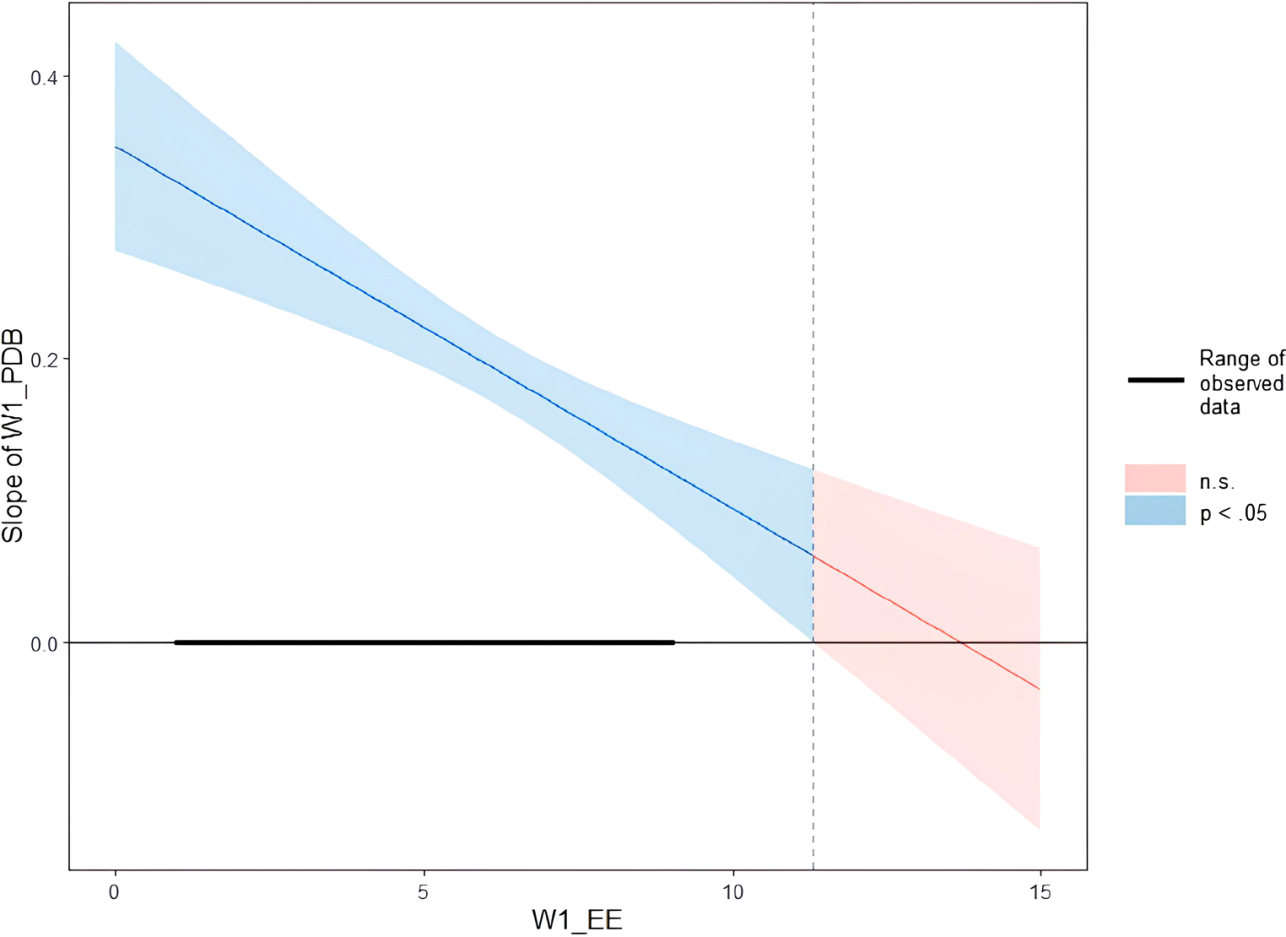
The moderating effect of educational expectations (Johnson–Neyman plot). EE, educational expectations; PDB, peer deviant behaviours; W1, Wave 1.

Table 4 shows the conditional indirect effects under different levels of educational expectations. First, the index of moderated mediation was significant (95% CI did not include 0) (Estimate = .033, *SE* = .016, 95% CI = [.003, .065], standardized estimate = .002), indicating that educational expectations significantly moderated the indirect effect of peer deviant behaviours on academic performance through one's own deviant behaviours. Second, the moderated mediation pattern was consistent with our Hypothesis 5. When the educational expectations were high (mean + 1 SD), the negative mediation effect of deviant behaviours on the link between peer deviant behaviours and academic performance was significantly weaker than when the educational expectations were low (mean − 1 SD) (standardized estimate contrast _high vs. low_ = .003, *p* < .05). Overall, adolescents' educational expectations could significantly reduce the mediation effect of their deviant behaviours on the association between peer deviant behaviours and academic performance.

**TABLE 4:** Conditional indirect effects.

Path	W1 EE conditions	Estimate	*SE*	LL	UL	Standardized estimate
W1_PDB → W2_DB → W2_AP	‐1SD	−.341	.064	−.474	−.223	−.009
	Mean	−.288	.053	−.400	−.190	−.007
	+1SD	−.235	.054	−.351	−.140	−.006
Index of moderated mediation		.033	.015	.003	.065	.002

Abbreviations: LL, 95% bootstrap confidence interval lower limit of (unstandardized) estimate; UL, 95% bootstrap confidence interval upper limit of (unstandardized) estimate.

### Supplementary analyses

Additional analyses were conducted to examine the robustness of the present findings. First, apart from our focal moderator (i.e., adolescent educational expectations) as an internal protective factor, we added extra moderators, family social capital indicators (as outlined above), as typical environmental protective resources to our moderated model. The results showed that specifying each of the family social capital indicators as an additional moderator did not change the current finding of the significant moderating effect of adolescent educational expectations, with the relative moderating effects of family social capital indicators non‐significant. Second, we alternatively employed the full information maximum likelihood (FIML in Mplus 8.0) to address the missingness, and the key results are similar to the current findings. For example, the moderation effect was significant (*β* = −.032, *p* < .01), and the mediation effect at the high level of educational expectations (standardized estimate = −.007, *p* < .001) was weaker than that at the low level (standardized estimate = −.009, *p* < .001). Third, as mentioned above, because there is no measure at W1 for deviant behaviours corresponding to that at W2 in the CEPS, we did not control for W1 deviant behaviours in the present analysis. To mitigate the potential impact of this constraint, we tried to indicate W1 deviant behaviours with one item (‘skipping class’) at W1 (overlapping with Item 7, ‘skipping class, truancy’ in the deviant behaviour measure at W2). By testing alternative models with this item additionally controlled, the results did not show noticeable differences from the current findings. These results support the robustness of our conclusions. The results of the supplementary analysis are available upon request.

## DISCUSSION

This research is among the first to examine the longitudinal moderated mediation mechanisms involving how educational expectations alter the links between peer deviant behaviours, adolescents' deviant behaviours and academic performance with a nationally representative sample in China.

Our findings indicate that peer deviant behaviours significantly undermined academic performance among Chinese adolescents when controlling for prior academic performance, family social capital, and various demographic factors. This aligns with our prediction based on social contagion theory (Levy & Nail, 1993) and previous research suggesting that deviant peer associations disrupt academic achievement (Jiang, 2023; Rambaran et al., 2017). Moreover, the mediating role of adolescents' deviant behaviours further supported our predictions based on social learning theory (Bandura, 1977) and coercion theory (Patterson, 1982). Specifically, exposure to deviant peers increases adolescents' deviant behaviours, which then negatively impacts academic performance. Our longitudinal findings extend the previous cross‐sectional findings (Jiang, 2023), providing cross‐time evidence of this mediation mechanism.

In particular, the path from peer deviance (W1) to adolescents' own deviance (W2) exhibits modest yet statistically significant effects, which is similar to previous longitudinal studies in China (Lin et al., 2020; Zhu et al., 2017) and other cultures (e.g., the United States) (Lansford et al., 2014; Wang & Dishion, 2012; see also Gallupe et al., 2019; Giletta et al., 2021, for meta‐analyses), indicating the potential cross‐cultural commonality of the effect of peer deviant behaviours on adolescent own deviance. Meanwhile, the path from adolescents' own deviant behaviours (W2) to academic performance (W2) is significant yet small in magnitude (with a standardized coefficient close to −.050). This estimate is less pronounced than some previous Western and Chinese findings (e.g., Jiang, 2023; McIntosh et al., 2008) but is closer to other research from both cultural contexts (e.g., Algozzine et al., 2010; Yu et al., 2022), revealing no clear evidence of cultural differences. The size of the indirect effect, whereby deviant behaviour mediates the relationship between peer deviance and adolescents' academic performance (standardized estimate = −.009), is generally small. This is expected, as noted earlier, since the second‐stage effect of the mediation chain is relatively modest in size. Compared to the literature, the indirect effect observed in our study is smaller than the effect reported by Jiang (2023), which was also derived from CEPS but based on a cross‐sectional design. The weaker mediation effect in our study could thus be attributed to the longitudinal design capturing the lessening (indirect) effect of peer deviant behaviour over time, given that the autoregressive effect of academic performance at the previous time point has been controlled. While a direct comparison is constrained by the limited existing longitudinal studies investigating this specific indirect effect, the present finding aligns with the broader theoretical view that peer influence is dynamically mediated through one's own conduct over time (Chen et al., 2008).

Educational expectations emerged as a significant protective factor, mitigating the impact of peer deviant behaviours on Chinese adolescents' deviant behaviours. This buffering effect aligns with the self‐determination theory (Ryan & Deci, 2000) and future time perspective theory (Lens et al., 2012), suggesting that fostering strong educational aspirations among adolescents could help minimize the negative peer effects. Importantly, the buffering effect of educational expectations remained evident even after accounting for family social capital indicators, with the relative moderating role of the latter non‐significant. This indicates that adolescents' own educational expectations contribute uniquely to their resilience against deviant peer influence over these family relational resources. Such a finding resonates with some previous research in the broader literature suggesting that individuals' internal psychological resources (e.g., individual agency, purpose in life, resilience attributes) may compensate for or override the familial contextual factors in shaping one's adjustment outcomes (e.g., Gong et al., 2024; Schoon & Cook, 2021; Zhou et al., 2024).

However, despite the significant buffering effect of educational expectations, the Johnson–Neyman analysis revealed that the buffering effect has limits. Even at the highest observable level of educational expectations, corresponding to ‘doctoral degree’, educational expectations could not completely neutralize the influence of peer deviant behaviours.

Such a moderating pattern aligns with the PRM (rather than PSM) (Xie et al., 2018), suggesting a need for caution in interpreting the (partial) protective role of educational expectations. Therefore, it might not be advisable to merely rely on such a single protective factor to address the adverse influence of deviant peers. Expanding adolescents' positive expectations to a broader range of life domains beyond education, including career development, interpersonal relationships and self‐actualization, may provide more comprehensive protective effects. Moreover, apart from internal protective resources like educational expectations, external resources such as teacher support and a positive school climate could complement efforts to mitigate the impacts of deviant peer behaviours, which could be examined by future research. Even so, this study demonstrates a robust protective effect of adolescent educational expectations, even after controlling for the potential environmental protective factors of family social capital indicators, with the latter not showing significant relative moderating effects. This underscores the potent role of educational expectations as an internal protective asset of adolescents.

Interestingly, educational expectations did not moderate the second stage of the mediation process (the link between adolescents' own deviant behaviours and academic performance). This suggests that once adolescents have already engaged in deviant behaviours, their educational expectations may no longer effectively protect their academic performance. Therefore, prevention and early interventions fostering educational aspirations (before deviant behaviours become entrenched) are important.

One potential avenue for the prevention and early intervention practices is to enhance awareness of education's long‐term value and to foster students' growth mindset at an initial stage (Yeager et al., 2019), such as the point at which students first enrol in the school. Meanwhile, fostering fairness and equity in the educational system may also help improve students' educational expectations and prevent deviant behaviours (Buchmann & Park, 2009; Parker et al., 2016). Policies could include expanding access to advanced educational programs for underrepresented students (Posselt, 2016), implementing equitable resource allocation to improve outcomes for disadvantaged students (Jackson et al., 2016), and ensuring an equitable distribution of qualified teachers through targeted recruitment and retention initiatives (Boyd et al., 2011; Darling‐Hammond, 2015). These efforts would strengthen protective institutional environments that may reduce vulnerability to deviant affiliation.

Some limitations should be acknowledged. First, while our two‐wave design establishes temporal precedence, longer‐term longitudinal studies with more intensive investigations would better capture the developmental trajectory of peer influence processes. Second, although CEPS provided objective data from the schools for academic performance, other measures, like deviant behaviours and deviant affiliation, relied only on participants' self‐reports. Adopting more objective measures in the future can help eliminate the interference of social desirability in responses. Third, we focused solely on the moderating role of students' educational expectations, which represent the most relevant aspect of future expectations for academic performance. Future research can further investigate whether expectations in other domains, such as career aspirations, may show similar protective implications. Last, although educational expectations are found to be a protective factor in this study, it is noteworthy that excessively high or unrealistic expectations may backfire in some contexts. As noted above, high educational expectations might be associated with elevated academic pressure, particularly in the Chinese context, which may in turn exacerbate adolescents' depressive symptoms (Zhou et al., 2021). Recent evidence also suggests that the implications of educational expectations for adolescent well‐being may depend on how such expectations align with the broader family context, as parent–child expectation discrepancies may lead to greater internalizing problems among adolescents (Ding et al., 2024). Future research can distinguish between adaptive and maladaptive forms of educational expectations, examining the potential impacts of overly elevated or misaligned expectations on adolescents' psychological strain, behavioural maladjustment and academic outcomes.

## CONCLUSION

This research revealed the negative longitudinal effect of peer deviant behaviours on Chinese adolescents' academic performance and delineated the moderated mediation mechanisms. In particular, educational expectations mitigate the effect of peer deviant behaviours on adolescents' own deviant behaviours, thus weakening the mediation effect of adolescents' deviant behaviours in the link between peer deviant behaviours and adolescents' academic performance. Our findings contribute to a more nuanced understanding of the protective role of educational expectations by highlighting both their strengths and limitations (i.e., boundary conditions), thereby informing school practitioners' efforts to design more targeted and effective educational interventions.

## AUTHOR CONTRIBUTIONS

**Yu Sun:** Conceptualization; data curation; formal analysis; writing – original draft. **Kaiji Zhou:** Methodology; formal analysis; writing – original draft. **Jacky C. K. Ng:** Conceptualization; data curation; supervision; writing – review and editing.

## FUNDING INFORMATION

This work was supported by Mini‐seed Fund from Mental Health Research Centre (Ref: P0048889), Faculty Collaborative Research Scheme between Social Sciences and Health Sciences from The Hong Kong Polytechnic University (Ref: P0054969) and Early Career Scheme from the Research Grants Council of Hong Kong (Ref: 25604623).

## CONFLICT OF INTEREST STATEMENT

The authors declare no conflicts of interest.

## Supporting information


**Appendix S1.** Item list for key measures used in this study.

## Data Availability

The data that support the findings of this study are available in the National Survey Research Center (NSRC) at http://ceps.ruc.edu.cn/, reference numbers are 72,810,330 (Wave 1) and 61,662,993 (Wave 2). These data were derived from the following resources available in the public domain: http://ceps.ruc.edu.cn/.

## Footnotes

^1^
There is no corresponding measure for the deviant behaviours at Wave 1 comparable with the 10‐item scale in Wave 2.
