# Supplementary material for: How and when do peer deviant behaviours link to adolescent academic performance?: Longitudinal moderated mediation analysis using a Chinese national sample
Source: Br J Educ Psychol. 2026 May 29;96(4):1479–500. doi: 10.1111/bjep.70097 (PMC13752670; doi:10.1111/bjep.70097)
Supplement: Supplementary file 1 [file BJEP-96-1479-s001.docx]

**Appendix A. Item list for key measures used in this study**

| **Variable** | | **Scale items** | **Response options** |
| --- | --- | --- | --- |
| **Peer deviant behaviors (W1)** | **Are any of your close friends doing the following activities?**  Skipping class, truancy  Violating school rules, being disciplined  Fighting  Smoking, drinking  Often going to internet cafes, game rooms  Engaging in romantic relationships  Dropped out of school | | 1 = None of them  2 = One or two of them  3 = Most of them |
| **Deviant behaviors**  **(W2)** | **Have you done the following behaviors?**  Cursing, using foul language  Quarreling  Fighting  Bullying weaker classmates  Losing temper easily  Difficulty concentrating  Skipping class, truancy  Copying homework, cheating on exams  Smoking, drinking  Going to internet cafes, game rooms | | 1 = Never  2 = Occasionally  3 = Sometimes  4 = Often  5 = Always |
| **Parent-child interaction (W1)** | **How often do you and your parents do the following activities?**  Having dinner  Reading books  Watching TV  Doing sports  Visiting museums, zoos, science centers  Going out for movies, shows, sports games | | 1 = Never  2 = Once a year  3 = Twice a year  4 = Once a month  5 = Once a week  6 = More than once a week |
| **Parent-child discussion (W1)** | **Do your parents often discuss the following issues with you?**  Things happening at school  Your relationships with friends  Your relationships with teachers  Your feelings or mood  Your worries or troubles  (Assessed for father and mother separately) | | 1 = Never  2 = Occasionally  3 = Often |
| **Parental engagement (W1)** | **During the past week, how often did your parents supervise your studies?**  Check your homework  Help with your schoolwork | | 1 = Never  2 = 1-2 days  3 = 3-4 days  4 = Almost every day |
| **Parent-child relationship** | **How is the general relationship between you and your parents?** | |  |
| **(W1)** | Relationship between you and your mother  Relationship between you and your father | | 1 = Not close  2 = Not too close nor too far  3 = Very close |
| **Parental expectations**  **(W1)** | **What is your parents’ requirement for your academic record?** | | 1 = No requirement  2 = About the average  3 = Above the average  4 = Top 5 in the class |
